# SARS-CoV-2 Virus-like Particles with Plasmonic Au Cores and S1-Spike Protein Coronas

**DOI:** 10.1021/acssynbio.3c00133

**Published:** 2023-07-14

**Authors:** Weronika Andrzejewska, Barbara Peplińska, Jagoda Litowczenko, Patryk Obstarczyk, Joanna Olesiak-Bańska, Stefan Jurga, Mikołaj Lewandowski

**Affiliations:** † NanoBioMedical Centre, Adam Mickiewicz University, Wszechnicy Piastowskiej 3, 61-614 Poznań, Poland; ‡ Institute of Advanced Materials, Wroclaw University of Science and Technology, Wybrzeże Wyspiańskiego 2, 50-370 Wrocław, Poland

**Keywords:** SARS-CoV-2, virus-like particles (VLPs), gold, localized surface plasmon resonance (LSPR), Raman spectroscopy, fluorescent imaging

## Abstract

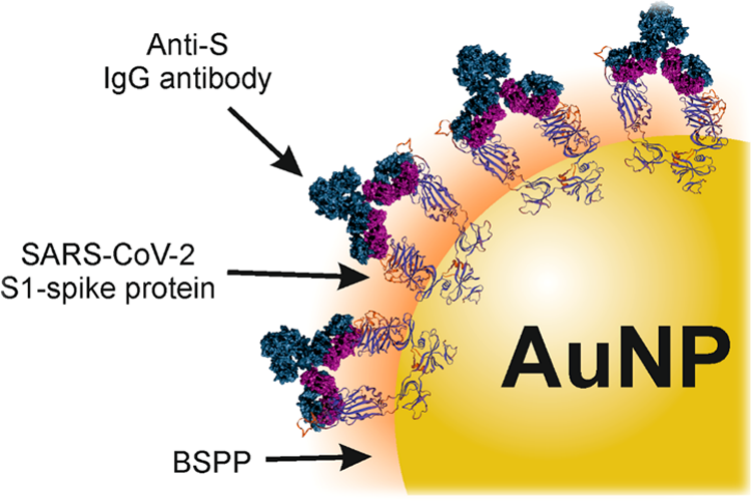

The COVID-19 pandemic has stimulated the scientific world to intensify virus-related studies aimed at the development of quick and safe ways of detecting viruses in the human body, studying the virus–antibody and virus–cell interactions, and designing nanocarriers for targeted antiviral therapies. However, research on dangerous viruses can only be performed in certified laboratories that follow strict safety procedures. Thus, developing deactivated virus constructs or safe-to-use virus-like objects, which imitate real viruses and allow performing virus-related studies in any research laboratory, constitutes an important scientific challenge. Such species, called virus-like particles (VLPs), contain instead of capsids with viral DNA/RNA empty or synthetic cores with real virus proteins attached to them. We have developed a method for the preparation of VLPs imitating the virus responsible for the COVID-19 disease: the SARS-CoV-2. The particles have Au cores surrounded by “coronas” of S1 domains of the virus’s spike protein. Importantly, they are safe to use and specifically interact with SARS-CoV-2 antibodies. Moreover, Au cores exhibit localized surface plasmon resonance (LSPR), which makes the synthesized VLPs suitable for biosensing applications. During the studies, the effect allowed us to visualize the interaction between the VLPs and the antibodies and identify the characteristic vibrational signals. What is more, additional functionalization of the particles with a fluorescent label revealed their potential in studying specific virus-related interactions. Notably, the universal character of the developed synthesis method makes it potentially applicable for fabricating VLPs imitating other life-threatening viruses.

## 1 Introduction

Since the beginning of the COVID-19 (coronavirus disease 2019) pandemic, scientists around the world have been intensively studying the virus responsible for the disease: the SARS-CoV-2 (severe acute respiratory syndrome coronavirus 2), trying to determine its structure and biological properties (such as the molecular mechanisms of human infection, cellular targets, and life cycle).^1^ Gaining this information is necessary for developing effective and rapid virus detection methods at the early stage of infection, as well as inventing new medicines and new-generation vaccines. However, conducting research with the use of infectious viral particles (even with inactivated capsids) is related to a potential health risk. Therefore, such studies can only be performed in scientific laboratories with the highest class biosafety (biological safety levels 3 and 4).^2,3^ This problem is addressed by the idea of using virus-like particles (VLPs)—noninfectious and safe-to-use biomimetic species that resemble certain features of a real viral molecule.^4^ VLPs can be used in vaccines, serve as virus phantoms, vehicles for targeted delivery of different materials (genes, peptides, drugs), and bioimaging contrast agents.^5^ One type of VLPs are those consisting of synthetic metallic cores and the surrounding protein “coronas”.^6^ The cores of such particles are usually characterized by potentially-applicable physical and chemical properties, while the coronas constitute bioactive layers and reduce the surface free energy of the cores.

In this work, we describe the method for synthesizing VLPs imitating the SARS-CoV-2. Au nanoparticles (AuNPs) with the size of ∼90–100 nm—similar to that of SARS-CoV-2’s capsid^7^—constitute the cores of VLPs. Gold is often utilized in biosensing applications due to its unique optical, electronic, and catalytic properties.^8−10^ Moreover, AuNPs specifically interact with various biomolecules, e.g., antibodies,^11,12^ proteins,^6,13^ and nucleic acids,^14^ which constitutes the basis of many virus detection systems. When surface-modified AuNPs are introduced into the solution of protein molecules, coronas rapidly form at their surface through chemical and physical interactions (such as van der Waals forces, hydrogen bonds, coordination, electrostatic or hydrophobic effects, as well as steric hindrance).^15^ The coronas of our VLPs are formed by S1 domains of the SARS-CoV-2 spike protein (the “S” protein). This domain was chosen because of its affinity to the ACE2 (angiotensin converting enzyme 2) receptor, which is located at the surface of cells prone to infection by SARS-CoV-2^16^ and mediates the membrane fusion for cell entry.^17,18^ The additional advantage of using Au-based VLPs is that they exhibit the so-called localized surface plasmon resonance (LSPR), which is a coherent and nonpropagating oscillation of free electrons in metallic objects subjected to an electromagnetic wave of an appropriate frequency (resonance frequency).^19,20^ Usually, the LSPR is excited with the use of light with a specific wavelength. The oscillation creates a strong electric field around the particle,^21^ which can, for example, enhance Raman scattering signals originating from species located in the vicinity of the nanoparticles (leading to the so-called surface-enhanced Raman scattering (SERS)).^22^ In the case of protein-covered particles, the LSPR—due to its sensitivity to the dielectric environment—can allow detecting specific interactions between proteins and antibodies.^23^ Our SARS-CoV-2 VLPs are the first to exhibit the LSPR effect. Through SERS, we were able to visualize the interaction between the VLPs and SARS-CoV-2 monoclonal antibodies (mAbs), which may constitute the basis for the future development of an LSPR-based COVID-19 test. Moreover, the functionalization of VLPs with fluorescently labeled antibodies revealed their potential in studying virus–cell interactions and designing targeted antiviral therapies.

## 2 Results and Discussion

### 2.1 Synthesis and Structural Characterization of SARS-CoV-2 VLPs

**Figure 1:**
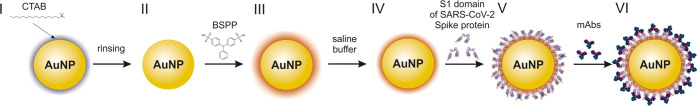
General scheme of the preparation of SARS-CoV-2 VLPs consisting of ∼100 nm Au cores and S1-Spike protein coronas.

Figure 1 illustrates the general scheme of the preparation of SARS-CoV-2 VLPs, divided into six steps. The synthesized AuNPs were initially coated with cetyltrimethylammonium bromide (CTAB) to prevent their aggregation (Figure 1, step I). Then, they were washed (step II) and CTAB was replaced with bis(*p*-sulfonatophenyl)phenylphosphine dihydrate dipotassium salt (BSPP) (step III). BSPP is a stabilizing agent^24^ and a surfactant necessary for further functionalization of AuNPs with the S1 domain of the S-protein. Optimization of the BSPP coating procedure was performed based on the literature protocols.^25−27^ Phosphines bind to Au through a lone phosphorus electron pair.^28^ Moreover, the chemical bonds between phosphines and Au are stronger than the electrostatic interactions between citrate or alkyl halides and Au, which allows easy ligand exchange. Additionally, BSPP is less toxic than CTAB, which is an important factor from the perspective of potential biomedical applications. The excess of BSPP was washed out with phosphate-buffered saline (PBS; step IV). Next, the BSPP-coated particles were dispersed in an optimized water solution of the S1 domain of the SARS-CoV-2 spike protein (step V). The free sulfonic groups of BSPP interact with the protein accounting for the formation of a corona layer.^29^ Finally, the as-prepared VLPs were incubated in a solution of anti-SARS-CoV-2 spike S1 mAbs to confirm their biological activity through a specific interaction (step VI).

To confirm the presence of S1 domains at the surface of AuNPs, sodium dodecyl-sulfate polyacrylamide gel electrophoresis (SDS-PAGE) was performed. The results are shown in Figure S1. As expected, based on the structural model and the information obtained from the manufacturer, under reducing conditions the protein is characterized by a molecular mass of 92 kDa. For the tested series of VLPs, a band corresponding to the presence of S1 domains appeared at a concentration of about 2 μg/mL, proving its presence at the surface of the studied particles. Notably, the intensity of the band was not changing significantly with increasing concentration, which indicates that 2 μg/mL constitutes the amount of protein that is needed to completely cover the surface of AuNPs.

**Figure 2:**
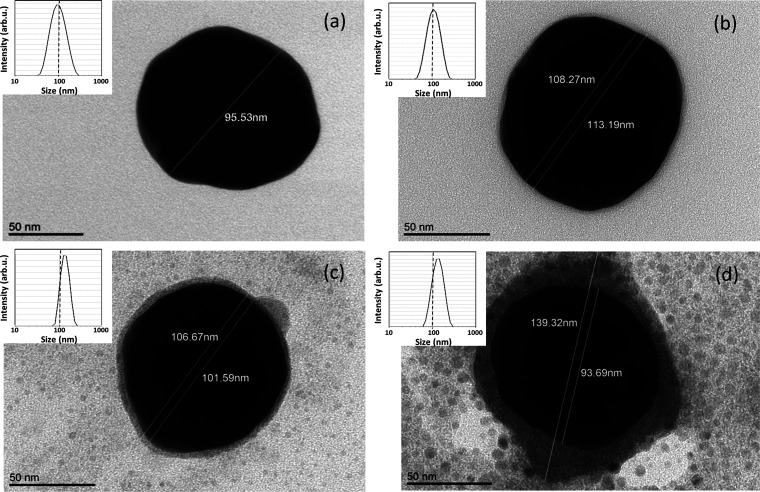
TEM images (a–d) and DLS size distribution histograms (insets) obtained at different stages of SARS-CoV-2 VLP preparation: AuNPs after removing the CTAB (a), the particles following the addition of BSPP (b) and AuNPs/BSPP after the attachment of S1 proteins (c). Panel (d) shows the VLPs following the exposure to SARS-CoV-2 anti-S mAbs. The measured diameters correspond to Au cores (smaller values) and cores with protein corona shells (bigger values).

Structural characterization of VLPs was performed using transmission electron microscopy (TEM), scanning electron microscopy (SEM), and dynamic light scattering (DLS) after the most important steps of the VLPs synthesis, i.e., II, IV, V, and VI. Additional measurements were carried out after the interaction of VLPs with mAbs (step VI), which was aimed at confirming their biological activity. The obtained TEM images are presented in Figure 2a–d, while the corresponding size distributions obtained from DLS are shown in the insets. SEM images recorded at steps II and V are presented in Figure S2. As can be seen from Figure 2a, the method used for the synthesis of AuNPs allowed obtaining spherical particles with an average size of ∼90–100 nm (measured without CTAB) and a narrow size distribution (93.01 ± 1.68 nm; polydispersity index (PDI) of 0.19). The image recorded after exchanging CTAB with BSPP is shown in Figure 2b, where BSPP is visible as a thin gray shell around the nanoparticle. DLS confirmed the ligand exchange by showing an increase in the average particle size to 103.4 ± 8.03 nm (PDI = 0.24). BSPP is known to form coordination complexes with AuNPs, which renders their high stability and provides protection against aggregation in water solutions.^30^Figure 2c shows a TEM micrograph obtained following the attachment of S1 proteins to the AuNP cores. A corona formed by protein molecules, which appear as dark species with a diameter of about 3 nm, is clearly visible. The recorded size-intensity distribution reveals the mean VLP size of about 124.50 ± 18.62 nm (PDI = 0.22). The high standard deviation is related to the thickness variation of the corona layer on different particles (ranging from 3 to 10 nm). A TEM image obtained after the incubation with anti-S mAbs is presented in Figure 2d. Compared to pure VLPs, a significant increase in the thickness of the corona layer was observed, up to 25 nm. The increase was also visible in DLS, which showed an average particle diameter of 175.24 ± 20.41 nm (PDI = 0.30). Thus, the performed experiment confirmed the biological activity of fabricated VLPs.

### 2.2 Surface Charge Analysis

The change of the surface charge may constitute an indirect proof of successful surface modification. Therefore, the surface charge at different stages of VLPs synthesis was determined from ζ-potential measurements and the results are summarized in Figure S3a. For AuNPs in a water solution of CTAB, a value of 46.85 ± 3.60 mV was determined. The high surface charge of AuNPs/CTAB leads to electrostatic repulsion between particles, which prevents their aggregation. After washing the particles out and dissolving in Milli-Q water, the surface charge dropped to 23.96 ± 3.80 mV. When the particles were further functionalized with BSPP, sulfonic groups imparted negative charges to the surface of AuNPs,^24^ which led to a similarly high but negative value of the surface charge (equal to −44.42 ± 3.67 mV). The obtained values indicate the importance of prompt replacement of one stabilizing and dispersing agent with the other (in this case CTAB with BSPP) in order to prevent AuNP aggregation. The formation of an S1 protein corona changed the ζ-potential to −10.00 ± 1.75 mV, which is slightly lower compared to the average value of native virions of the coronavirus family (−25.68 mV).^31^ The VLP/mAb complexes were characterized by a similar surface charge value of −12.25 ± 1.95 mV. These low values obtained for VLPs and VLPs/mAbs may account for their tendency to aggregate, as observed on SEM images (Figure S2). Most importantly, the evolution of the ζ-potential at different stages of VLP synthesis was found to be in line with the morphological changes observed with TEM.

### 2.3 Spectroscopic Characterization

**Figure 3:**
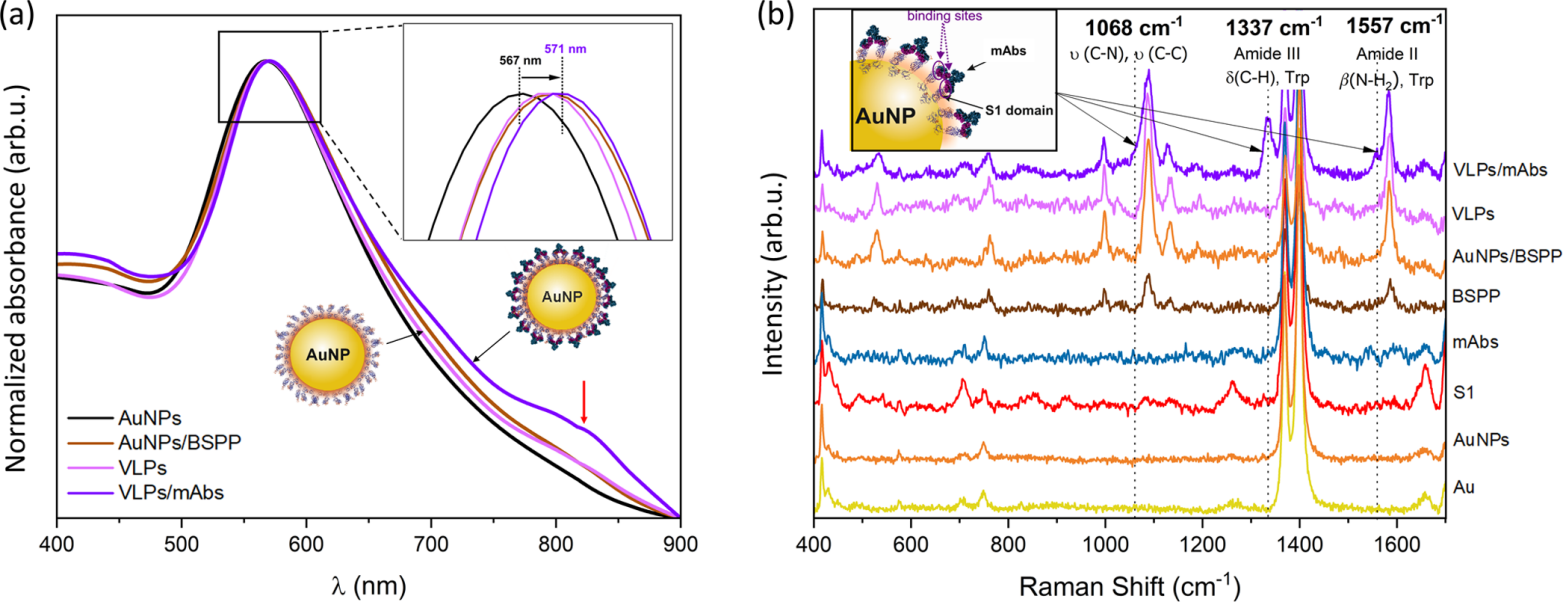
UV–Vis (a) and Raman (b) spectra obtained at different stages of the SARS-CoV-2 VLPs preparation and after exposing the VLPs to SARS-CoV-2 anti-S mAbs. UV–Vis reveals the shifts in the LSPR signal related to the attachment of the molecules to the surface of AuNPs. Raman spectroscopy allows the identification of peaks related to the interaction of VLPs with mAbs (bands at 1068, 1337, and 1557 cm^–1^, originating from aromatic amino acids—Trp and Phe).

Spectroscopic characterization of VLPs was performed using UV–Vis and Raman spectroscopy. The UV–Vis absorption spectrum of pristine AuNPs shows a strong LSPR peak centered at around 567 nm (Figure 3a). The position of this peak is related to the size of the particles, their spherical shape, and the refractive index of the medium (water). After covering the particles with BSPP, the position of the peak red-shifts by 3 nm, which is typical for surface-modified Au species.^32^ The resonance shift results from the change in the dielectric environment of AuNPs related to the attachment of molecules.^33^ Following the addition of S1 proteins, no further shift is observed, which is due to the presence of a relatively thick BSPP layer and the associated negligible influence of additional molecules on the LSPR excited at the Au surface (as even a significant change in the dielectric environment taking place several nanometers from the surface does not influence the LSPR in gold^34−37^). Similarly, no shift (within the limit of error, i.e., 1 nm) is observed when combining VLPs with mAbs. However, it has to be noted that the signal recorded for the VLP/mAb complexes is much broader, exhibiting a shoulder at around 800 nm (marked with a red arrow in Figure 3a). This change in the signal is related to the formation of VLP/mAb agglomerates of different sizes that give rise to a family of red-shifted absorption signals (which account for the broadening). Another important aspect from the point of view of potential applications of VLPs, is their temporal stability. Figure S3b reveals that the position of the LSPR peak does not change after 10 days from VLPs preparation and its intensity does not decrease. This confirms that the fabricated particles are temporarily stable.

Raman spectroscopy measurements allowed getting insight into the interaction of VLPs with the SARS-CoV-2 anti-S mAbs through the analysis of vibrational signals. The results are shown in Figure 3b. The presence of LSPR in gold leads to the amplification of Raman peaks coming from molecules residing at the surface of AuNPs (the appearance of the SERS effect).^38,39^ This facilitates the identification of characteristic vibrational signals of the S1 protein and mAbs, as well as the observation of changes in these signals resulting from the VLPs–mAbs interaction. For the studies, the solution of VLPs was drop-casted onto a 12 nm-thick Au film deposited onto an α-Al_2_O_3_(0001) (ALO) single-crystal substrate. The selection of the substrate was not incidental, as gold films deposited onto dielectric substrates enhance the SERS effect.^40,41^ In addition to VLPs and VLP/mAb complexes, measurements were also performed for clean Au/ALO, as well as AuNPs, AuNPs/BSSP, S1 proteins, and mAbs, deposited from solutions onto Au/ALO. In the case of a clean Au/ALO substrate, bands located at 416, 428, 448, 488, 575, 706, 748, 826, 1266, 1370, 1400, and 1657 cm^–1^ were observed (Table S1). Higher-frequency peaks (>1000 cm^–1^) were expected to appear for Au, while the intensity of peaks positioned at lower frequencies (<1000 cm^–1^) was found to decrease with an increase in the thickness of the deposited gold layer (not shown), due to which these peaks were assigned to originate from ALO.^42,43^ Only one additional band, located at 496 cm^–1^, was observed after drop-casting pure AuNPs onto the Au/ALO substrate. This peak is most probably related to the presence of sulfur (the S–S vibrational stretching mode^44^) adsorbed on gold from air. The spectrum obtained for S1 proteins contains numerous bands corresponding to vibrations within different molecular groups.^44−46^ The strongest peaks are observed at 494, 525, 542, and 556 cm^–1^ and originate from the stretching of S–S bonds.^44^ The bands at 621 and 642 cm^–1^ can be correlated with Phe and C–S stretching modes,^45^ respectively, the band at 781 cm^–1^ with the movements of the Tyr residues,^47^ while the one at 919 cm^–1^ with the C–C stretching. In the amide III area, vibrations at 1136 and 1433 cm^–1^—coming from CH/CH_2_ deformations—appear. Further, at 1516 cm^–1^, a signal from Trp is visible. Finally, at 1620 cm^–1^, a peak related to amide I can be noticed. In the case of SARS-CoV-2 anti-S mAbs, which belong to the immunoglobulin group (IgG), fewer bands were observed: at 491 cm^–1^ (S–S),^48^ ∼600 cm^–1^ (Phe),^44^ and 1164 cm^–1^ (alkyl C–N vibrations).^47^ The positions of these peaks are similar to those observed for S1 proteins, which is due to the presence of amino acids in the structure of both biomolecules. In addition, vibrations coming from CH/CH_2_ deformations (1455 cm^–1^),^48^ aromatic rings in Trp (at 1544 cm^–1^) and Phe (1600 cm^–1^), as well as the peak related to amid I band (1630 cm^–1^),^49^ were observed. The spectrum of Au/BSPP contains bands at 525, 623, and 697 cm^–1^, coming from the stretching S–S, C–P, and C–S modes, respectively.^50,51^ Due to the fact that BSPP belongs to phosphines, characteristic peaks were also observed at 997, 1032, and 1089 cm^–1^,^52^ as well as at 1481 cm^–1^ (CH/CH_2_ deformations) and 1585 cm^–1^ (benzene rings).^53,54^ The molecule also features two K–O bonds, the peaks of which were visible at 1132 and 1189 cm^–1^, as well as O=S=O groups, giving asymmetric stretching vibrations manifested by a peak located at 1273 cm^–1^.^55^ BSPP in combination with AuNPs gives a much clearer signal due to combined SERS from AuNPs and the gold substrate. The spectrum of VLPs contains bands originating from the attached S1 proteins—mainly those located at 493, 530, 621, 630, 780, and 913 cm^–1^. Thanks to the appearance of the SERS effect, two additional bands—at 757 and 977 cm^–1^—which did not clearly appear in the spectrum of pure S1 proteins, could be observed. The most significant and interesting changes in the spectra occurred after exposing VLPs to mAbs. As a result of the attachment of the antibodies to S1 proteins, three new bands appeared: at ∼1068, 1337, and 1557 cm^–1^. The first band lies at the conformationally-sensitive region of protein skeletal modes and can be associated with the stretching C–N and C–C vibrations in amino acids.^44,48^ It is known that protein–antibody interactions involve, in particular, the N-terminal groups of proteins.^56,57^ Therefore, the appearance of this band is attributed to the binding mechanism of the antibody to the S1 domain of the Spike protein (its RBD domain).^58^ The second new band, appearing at 1337 cm^–1^, falls within the amide III region, in which C–H deformation modes are observed. This area is often associated with Trp vibrations, which may also appear in the case of protein/antibody conjugates^44,45,47,48^ (as both the stacking interactions from aromatic amino acid rings^59^ and Trp are present in the binding of antibodies to proteins^60^). The third new band at 1557 cm^–1^ appears in the amide II region and comes from the NH_2_ bending motions,^34,61,62^ as well as from Trp.^44,45,47,48^ The newly identified bands related to the specific interaction of VLPs with mAbs may constitute the basis of SERS-based SARS-CoV-2 detection.

### 2.4 Fluorescent Imaging

**Figure 4:**
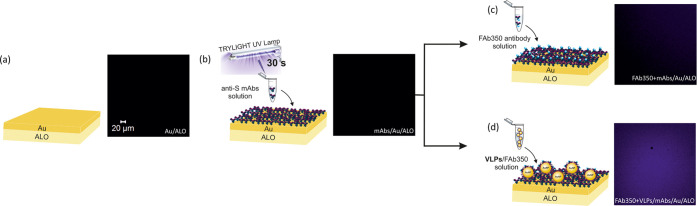
Scanning confocal microscopy images obtained for the Au/ALO substrate (a), Au/ALO surface-functionalized with nonfluorescent SARS-CoV-2 anti-SmAbs (b), the functionalized substrate after incubation with FAb350 (c) and after incubation with VLPs fluorescently labeled with FAb350 (d).

Finally, we have evaluated the potential of fabricated VLPs in fluorescent imaging. For this purpose, a Au/ALO substrate with immobilized anti-S mAbs was prepared by irradiating the mAbs solution with UV light for 30 s (total UV intensity on the cuvette: 3 W/cm^2^) and drop-casting the irradiated solution onto the gold film. UV irradiation is a well-established and effective immobilization procedure, as it promotes the photoreduction of S–S bridges in IgG mAbs through the activation of Trp/Cys–Cys triads, which may either recombine or bind to the neighboring Au species.^48^ The immunocomplex was then rinsed 3 times with PBS to detach unbounded FAb350. The confocal microscopy results showed no fluorescence from the Au/ALO and mAbs/Au/ALO substrates (Figure 4a,b). For the mAbs/Au/ALO incubated with FAb350, which was used as a negative control (as FAb350 should not bind to mAbs and give a fluorescent signal), low fluorescence was detected due to a possible nonspecific adsorption (Figure 4c). However, for mAbs/Au/ALO incubated with VLPs/FAb350, an intense and uniform fluorescent signal was observed (Figure 4d). This confirmed that the fluorescently labeled VLPs were able to react specifically with the antibodies (and potentially also other species, such as ACE2 receptors). The high intensity of fluorescence is most probably related to the extended incubation times of mAbs with Au/ALO, Fab350 with VLPs, and fluorescently labeled VLPs with the mAbs/Au/ALO substrate, which resulted in a high concentration of fluorescent species at the imaged surface. The fact that the intensity was uniform across the surface (with single VLPs not being visible) is most probably related to the above-mentioned saturation of the surface with fluorescent species and the limited resolution of the imaging instrument (which is lower than the size of a single VLP). Most importantly, the studies confirmed that the developed VLPs could be potentially used for fluorescent imaging.

## 3 Conclusions

SARS-CoV-2 virus-like particles were synthesized by attaching the S1 domains of real viruses’ Spike protein to the Au cores constituting the capsids. The particles were characterized with respect to their structure and optical properties, revealing the formation of S1 protein coronas around Au cores and the presence of localized surface plasmon resonance in the cores. They were also found to preserve their biological activity toward SARS-CoV-2 anti-S monoclonal antibodies. The interaction between the virus-like particles and the antibodies was monitored using Raman spectroscopy, and the appearance of three new vibrational signals, related to the binding of the two species, was observed. The identified characteristic vibrational signals of the S1 proteins and the anti-S mAbs may constitute the basis for Raman-based SARS-CoV-2 infection detection. Moreover, fluorescently labeled virus-like particles revealed their potential in fluorescent imaging. All this indicated that the fabricated particles are suitable for biosensing applications and studying virus–cell interactions. Notably, the developed preparation procedure, having a relatively universal character, can be potentially applied to fabricate virus-like particles imitating other life-threatening viruses.

## 4 Materials and Methods

### 4.1 Synthesis of AuNPs

Citrate-stabilized AuNPs (diameter 90 < *d* ≤ 100 nm) were prepared using the modified method of Rodríguez-Fernández et al.^63^ and Jana et al.^64^ (the so-called Turkevich method). In the first step, gold seeds (*d* = ∼10 nm) were synthesized. In brief, HAuCl_4_ aqueous solution (25 mL, 0.25 mM) was preheated to 100 °C and reduced by a fast injection of trisodium citrate aqueous solution (0.5 mL, 34 mM, also preheated to ∼100 °C) under vigorous stirring (1200 rpm). The reaction was carried out in a 50 mL round-bottom flask with a reflux condenser, which was switched on until the color of the solution changed to ruby red (30 min). The resulting product was purified two times via centrifugation at 18 000*g* for 45 min. Then, the supernatant was removed and the pellet was dispersed in the corresponding volume of a Milli-Q grade water. After initial purification, 5 mL of gold seeds was diluted with an equivalent volume of a 0.03 M CTAB aqueous solution, gently mixed, and left overnight under ambient conditions. UV–Vis spectra of the as-prepared seed solution revealed a peak at 523 nm. Subsequently, gold seeds were overgrown to obtain AuNPs with *d* = ∼100 nm. For this purpose, a new growth solution was prepared (0.125 mM HAuCl_4_, 0.015 M CTAB, and 0.5 mM (l)-ascorbic acid) and preheated to 50 °C. Afterward, 0.5 mL of gold seeds was injected into the growth solution, gently mixed by inversion, and left undisturbed for 2 h. In this process, in addition to AuNPs, Au species with other shapes—such as nanorods or nanospheres—are obtained. Thus, additional purification was carried out following the procedure reported by Jana et al.^64^ (in their work, the impurities were separated from the supernatant containing spherical nanoparticles with *d* = ∼100 nm). The resulting solution was purified by centrifugation (3000*g*, 5 min), redispersed in water (two times) and, finally, in a 0.01 M solution of CTAB (to be stored). All of the reagents were used as purchased, without further purification. The Milli-Q water was obtained using the Hydrolab DH-0005-UV purification system. Trisodium citrate dihydrate (Na_3_Cit) (≥99%), gold(III) chloride trihydrate (HAuCl_4_·3H_2_O, ≥99.9%), (l)-ascorbic acid (99%), and CTAB (≥99%, for biochemistry) were supplied by Sigma-Aldrich.

### 4.2 SARS-CoV-2 S1-Spike Proteins and anti-S mAbs

The recombinant SARS-CoV-2 spike S1 domain C-terminal 6-His tag proteins (Val16–Pro681) and the anti-S mouse IgG mAbs were purchased from Bio-Techne/R&D Systems and dissolved in 0.1 M PBS with a pH of 7.4. The concentration of the protein stock solution was determined by absorbance (*A*_280_) using NanoDrop2000c, with the theoretical molar extinction coefficient calculated using the ProtParam tool (ExPASy).^65^ The SDS-PAGE performed for 50 μg/mL of this protein showed a molecular mass of about 75 kDa (Figure S1).

### 4.3 Preparation of SARS-CoV-2 VLPs

The solution of AuNPs was rinsed by centrifugation and dispersed for stabilization. Then, the CTAB coating was replaced with BSPP. For this purpose, AuNPs in 30 mM CTAB solution were heated up to 40 °C in a sonic bath and centrifuged three times at 3000 RCF for 15 min to remove the surfactant. After two centrifugations, the supernatant was removed and AuNPs were dissolved in 1 mL of Milli-Q water. After the third centrifugation, AuNPs were dissolved in an earlier prepared BSPP (Merck Millipore) water solution (4 mg, 7.5 mM) and incubated for 6 h with shaking (850 RPM, 22 °C) in the dark. To remove the excess of BSPP, the samples were centrifuged at 300 RCF for 10 min. 500 μL of pellet was taken, and the rest was resuspended in an additional 500 μL of Milli-Q water. Finally, 500 μL of purified recombinant SARS-CoV-2 S1 protein in a concentration of 6.25 μg/mL was added to 500 μL of BSPP-coated AuNPs (4 × 10^9^ particles/mL). The mixture was then incubated for 1 h at room temperature.

### 4.4 Absorption Spectroscopy (UV–Vis)

The LSPR signal was recorded for the VLPs solution in standard 1 cm quartz cuvettes (Hellma Analytics) using a PerkinElmer Lambda 950 UV–Vis–NIR spectrometer in the range of 300–900 nm (W lamp) with a 0.1 nm resolution. The number concentration *N* of the particles was calculated based on the absorption according to formula 1
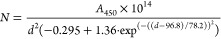
1where *A*_450_ is the adsorption measured at 450 nm and *d* is the diameter of the particles given by eq 2
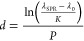
2Based on the Mie theory, for nanoparticles with a diameter larger than 25 nm, the appropriate fit parameters are *K* = 6.53, *P* = 0.0216, and λ_0_ = 512 nm.^66^

### 4.5 Determination of Particle Size Using DLS

The DLS measurements were performed with the use of a Malvern Zetasizer Nano ZS90 instrument equipped with a 633 nm He–Ne laser and a photodiode detector set at a 173° detection angle. The samples were equilibrated at 25 °C in a standard quartz cuvette. The refractive index of gold was set as 0.2 and the viscosity of the medium to that of water. The *Z*-average particle diameter with appropriate PDI was measured. An average of 10 measurements was used for the analysis.

### 4.6 ζ-Potential

The measurements were performed using the same instrument as the DLS, with the samples equilibrated at 25 °C in a ζ-potential standard cell.

### 4.7 Electron Microscopy

The TEM studies were performed using a JEOL JEM-1400 microscope with a 120 kV operating voltage. 10 μL of samples were dried for 1 min on a standard 300-mesh Cu grid with carbon Formvar (Agar Scientific). After drying, the remaining liquid was removed by touching the grid edge with a low lint paper and stained with 5 μL of 2% uranyl formate solution, which was removed after 1 min. SEM studies were carried out with the use of a JEOL JEM 7001F microscope with an SEI detector, using a 15 kV accelerating voltage.

### 4.8 Raman Spectroscopy

The Raman spectroscopy measurements were performed using Renishaw inVia instrument with the 633 nm He–Ne laser. The spectrometer grating conditions were 1800 grooves/mm and the acquisition time was 20 s with 1 accumulation. The beam was focused on the sample with a 20× microscope lens with a numerical aperture of 0.4. The measurements were performed in the backscattering geometry with a spectral resolution of 1.0 cm^–1^. The laser power was adjusted between 50 and 100% (*P*_max_ = 17 mW). All of the measurements were taken at room temperature, after drying the drop-casted samples onto an α-Al_2_O_3_(0001) substrate covered with a 12.7 nm-thick Au layer deposited from a crucible with the use of an electron beam evaporator (Telemark/PREVAC) under ultra-high vacuum (UHV). In order to extract the Raman signals of interest, the background was subtracted from the acquired raw spectra through the appropriate algorithm, and the data were analyzed using the OriginLab software.

### 4.9 Fluorescence Microscopy

Fluorescence-activated measurements were performed using a Zeiss LSM 780 confocal laser scanning microscope with a 40× water objective, an excitation laser wavelength of 405 nm, and an emission of 441 nm for detecting fluorescence. The measurements were repeated at least three times to obtain proper statistics.

### 4.10 SDS-PAGE

Freshly prepared solutions of VLPs with different concentrations of the S1 protein (from 1.6 to 6.3 μg/mL) were concentrated by centrifugation at 3000 RCF for 15 min at room temperature. Then, to extract the proteins from the surface of AuNPs, SDS-PAGE incubation buffer was added. After heating to 95 °C, to denature the proteins, and cooling on ice for 2 min, the samples were subjected to an SDS-PAGE gel. Two solutions of S1 protein with concentrations of 50 and 6.3 μg/mL were used as references. SDS-PAGE electrophoresis under reducing conditions was performed by adding incubation buffer to washed VLPs in a 1:1 ratio (15 μL:15 μL) and loading the sample into the gel lanes (30 μL/lane) (4–15% Mini-PROTEAN TGX Stain-Free Protein Gels, Bio-Rad). As a reference marker, Precision Plus Protein Unstained Standards (Bio-Rad) were used. The experiment was conducted at a constant voltage of 110 V. After electrophoresis, the gel was washed for 5 min in water, cleaned for 1 h in 15% ethanol and in 1% citric acid water solution, washed for 5 min in water, and stained overnight at 4 °C in Coomassie Blue staining reagent (Serva Electrophoresis GmBH, Germany). For visualization, a Pharos FX Plus Molecular Imager (Bio-Rad) was used.

## Supporting Information Available

Result of SDS-PAGE electrophoresis obtained for a series of SARS-CoV-2 VLPs, together with the structural model of SARS-CoV-2 spike protein (PDB 6VXX; Figure S1); SEM images of pristine AuNPs and the SARS-CoV-2 VLPs (Figure S2); ζ-potential values obtained for the studied AuNPs and their conjugates (Figure S3a); normalized UV–Vis spectra of pristine AuNPs, freshly synthesized SARS-CoV-2 VLPs, and the particles after 10 days from the preparation (Figure S3b); assignment of Raman spectroscopy peaks observed for Au/ALO, AuNPs, BSPP, S1, mAb, AuNPs/BSPP, VLPs, and VLP/mAb complexes (Table S1) (PDF)
