# Supplementary material for: SARS-CoV-2 Virus-like Particles with Plasmonic Au Cores and S1-Spike Protein Coronas
Source: ACS Synth Biol. 2023 Jul 14;12(8):2320–8. doi: 10.1021/acssynbio.3c00133 (PMC10443039; doi:10.1021/acssynbio.3c00133)
Supplement: Supplementary file 1 — sb3c00133_si_001.pdf [file sb3c00133_si_001.pdf]

Supporting Information

SARS-CoV-2 Virus-Like Particles with Plasmonic Au Cores and S1-Spike Protein Coronas

Weronika Andrzejewska,[#] Barbara Peplińska,[#] Jagoda Litowczenko,[#] Patryk Obstarczyk,[&] Joanna Olesiak-Bańska,[&] Stefan Jurga,[#] Mikołaj Lewandowski[#,*]

[#]*NanoBioMedical Centre, Adam Mickiewicz University, Wszechnicy Piastowskiej 3, 61-614 Poznań, Poland*

[&]*Institute of Advanced Materials, Wroclaw University of Science and Technology, Wybrzeże Wyspiańskiego 2, 50-370 Wrocław, Poland*

[*]*Corresponding author: lewandowski@amu.edu.pl*

Keywords: SARS-CoV-2, virus-like particles (VLPs), gold, localized surface plasmon resonance (LSPR), Raman spectroscopy, fluorescent imaging

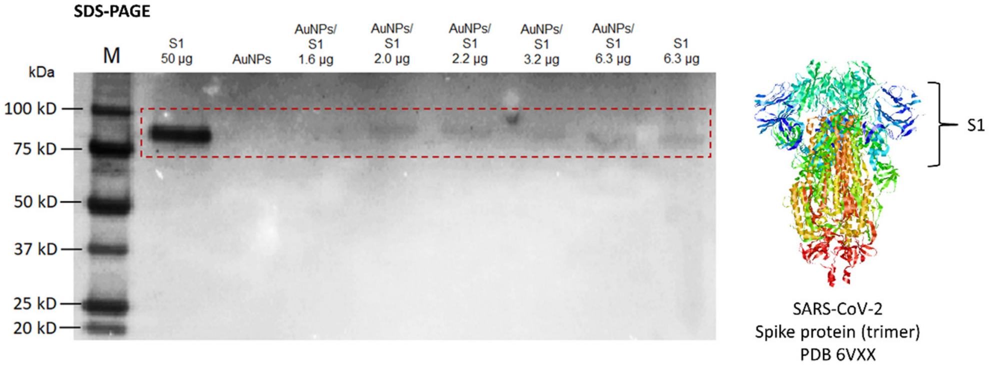

Figure S1. (Left) The result of SDS-PAGE electrophoresis obtained for a series of SARS-CoV-2 VLPs synthesized by incubating Au/BSPP cores in solutions of S1 proteins with different concentration per 1 mL. (Right) Structural model of the SARS-CoV-2 Spike protein (PDB 6VXX).[1]

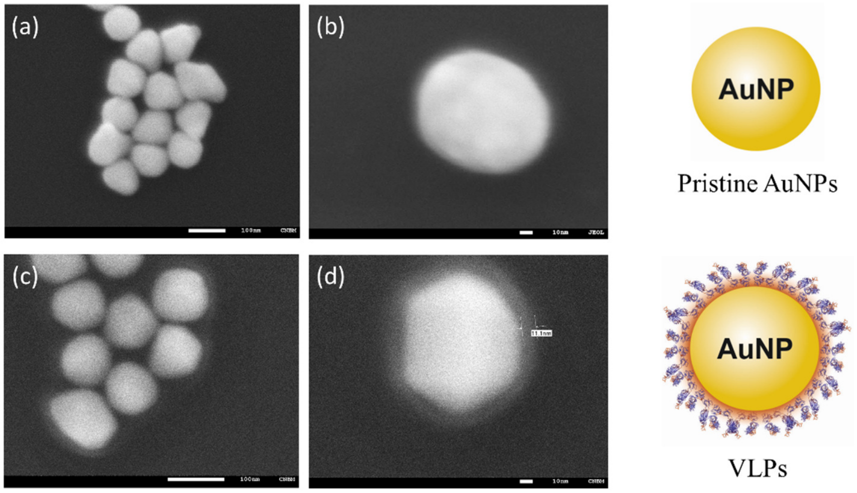

Figure S2. SEM images of pristine AuNPs (a,b) and SARS-CoV-2 VLPs (c,d). The corona of S1 proteins is clearly visible on (d). When the VLPs aggregate, their shells start to form a continuous matrix in which Au cores are embedded (c).

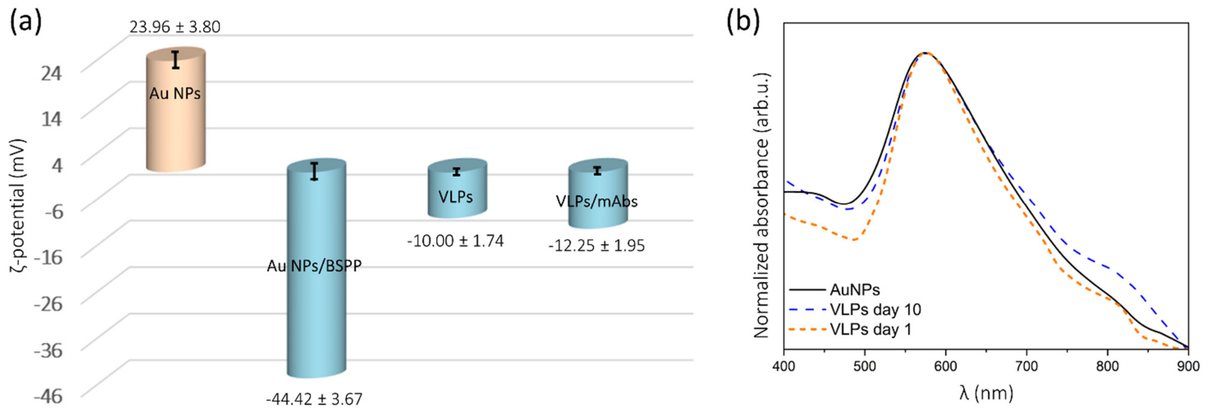

Figure S3. (a) ζ-potential values obtained for the studied AuNPs and their conjugates. (b) Normalized UV-Vis spectra of pristine AuNPs (black line), freshly-synthesized SARS-CoV-2 VLPs (orange) and VLPs after 10 days from preparation (blue).

Table S1. Vibrational modes (Raman spectroscopy peak positions, cm^{-1}) observed for Au/ALO, AuNPs, BSPP, S1 proteins, mAbs, AuNPs/BSPP, SARS-CoV-2 VLPs and VLP/mAb complexes.

Raman (cm^{-1})				SERS (cm^{-1})				Assignment
Au/ALO	BSPP	S1	mAbs	AuNPs	AuNPs/BSPP	VLPs	VLP/mAb complexes	
416	418	416	416	416	418	418	416	
428		430	430	428	428	428	428	
448		448	448	448	448	448	448	
488								
		494	491	496	490	493	492	$\nu(S\text{-}S)_{str}$
	525	525			525	530	531	$\nu(S\text{-}S)_{str}$
		542						$\nu(S\text{-}S)_{str}$
		556						$\gamma(NH_2)$, $\nu(S\text{-}S)_{str}$
575	577	576	577	575	576	576	576	
			600					*Phe*
		621				621		*Phe*, $\nu(C\text{-}S)_{str}$
	623		630		623	630	630	$\nu(C\text{-}P)$
		642						$\nu(C\text{-}S)$
	697		696		692			$\nu(C\text{-}S)$
706		706	710	706		708	708	
748		750	751	748				
						757	757	*Tyr, Trp*
		781				780		*Tyr*
826	843	848		826		832	832	
		919				913	913	$\nu(C\text{-}C)_{str}$
						977	977	$\nu(C\text{-}C)_{str}$
	997				997	999	996	*phosphine*
	1032	1038			1032	1025	1032	*phosphine*, $\nu(C\text{-}$
							**1068**	**$\nu(C\text{-}N)_{str}$, $\nu(C\text{-}$**
	1089				1089	1089		*phosphine*
	1132				1132	1132	1132	*K-O*

	1189				1189	1189	1189	*K-O*
		1136						*Amide III*
			1164					*alkyl ν(C-N)*
1266		1263	1248	1266		1266	1265	*Amide III*
	1273				1273	1273		*ν(S=O=S)*
							**1337**	***Amide III,***
1370				1370				
1400				1400				
		1433						*$\delta(CH,CH_2)$*
		1458	1455					*$\delta(CH,CH_2)$*
	1481				1480			*$\delta(CH,CH_2)$*
		1516	1544					Trp
							**1557**	**Amide II,**
	1585	1594	1590		1583			*ν(C-C), $\delta(NH_2)$*
			1600					*Phe*
		1620	1630				1623	*Amide I*
1658	1652	1659	1658	1658	1655	1656	1658	

The bands were assigned based on the literature (Refs. 38–62) (see the main text for details).

References

(1) Walls, A. C.; Park, Y. J.; Tortorici, M. A.; Wall, A.; McGuire, A. T.; Veesler, D. Structure, Function, and Antigenicity of the SARS-CoV-2 Spike Glycoprotein. *Cell* **2020**, *181*, 281–292.
